# An Interesting Meeting

**Published:** 1887-02

**Authors:** 


### EDITORIAL.

### An Interesting Meeting.

The annual meeting of the first District Dental Society, of New York, was held on the 17th, 18th, and 19th of January. It was a remarkable meeting in several respects. The attendance was very large; there were present about six hundred; probably a greater number than was ever present at any dental meeting in this country, and some ventured the opinion that such a collection of dentists has never been together in the world.

Monday and Tuesday evenings were devoted to the reading and discussion of papers. Quite a number of very good papers were read. From them and the discussion following, new ideas were derived, and thought stimulated. The following papers attracted special attention: "The Rational Basis of Practice," by Prof. James Truman, of the Dental Department of the University of Pennsylvania. Some of the positions taken in this paper elicited quite a warm discussion.

Dr. N. W. Kingsley presented a "Critical Essay on Treatment of Irregularities of the Teeth." In this essay some practitioners in this line were criticized severely, rather more so, we think, than the best interest of those present required. It is gratifying, however, to say that the practice and methods of our friend and neighbor, Dr. Geo. W. Keely, of Oxford, Ohio, were approved, endorsed by the essayist—perhaps he would hardly act otherwise to an Ohio man.

A very good paper was presented by Dr. J. Rollo Knapp, of New Orleans, on "Crown and Bridge Work." It was exhaustive and complete, presenting the process of crown work more fully and perfectly than we have seen it elsewhere done.

Dr. T. D. Shumway read a paper on "Science and Dentistry." In this the doctor endeavored to set forth in a plain and understandable manner some points that, as usually presented, are hedged about with more or less of mist and obscurity; for this he was somewhat criticized, nevertheless, his paper was a good one, and will be valuable for many, to whom a more abstruse presentation would be valueless. These are referred to merely as specimens of the papers read.

Two days, Tuesday and Wednesday, were devoted wholly to clinics, and the examination of new and valuable devices and instruments. Not the least important and pleasant employment of this time was the opportunity for social converse. This is a resource of our dental gatherings that has not been cultivated and utilized as it should have been. The way was open at this meeting for the accomplishment of more in this direction than we have ever seen before.

Would it not be well, and productive of great good, to have in every dental meeting consisting of any considerable number of persons, and especially where there are strangers, a committee whose duty it should be to promote social intercourse, and the personal acquaintance of all present?

It is quite impracticable here to attempt to give a detailed account of the clinics, and the work done in crowns and bridges.

The following are some of those who engaged in clinics: Miss S. E. Feltwell, E. P. Brown, Truman W. Brophy, C. F. W. Bodecker, R. Walter Starr, W. H. Atkinson, S. G. Perry, A. H. Parr, J. N. Farrar, S. H. Guilford, and many others. Only those who were present can realize what those who were absent missed. The meeting, upon the whole, was a grand success. To the officers and committees who devised and carried out the details of the meeting, the congratulations and thanks of all present are due, and will most certainly be accorded.

